# Subacute and Chronic Low-Back Pain: From MRI Phenotype to Imaging-Guided Interventions

**DOI:** 10.3390/diagnostics16020240

**Published:** 2026-01-12

**Authors:** Giulia Pacella, Raffaele Natella, Federico Bruno, Michele Fischetti, Michela Bruno, Maria Chiara Brunese, Mario Brunese, Alfonso Forte, Francesco Forte, Biagio Apollonio, Daniele Giuseppe Romano, Marcello Zappia

**Affiliations:** 1 Department of Medicine and Health Science “V. Tiberio”, University of Molise, 86100 Campobasso, Italy; 2 Fondazione Trotula de Ruggiero, 84121 Salerno, Italy; 3 Biomedical Research Centre, Gruppo Forte, 84124 Salerno, Italy; 4 Ospedale S. Salvatore, 67100 L’Aquila, Italy; 5 Centro Diagnostico Ricerche Radiologiche s.r.l., Via Pierluigi da Palestrina 1, 70056 Molfetta, Italy; 6 Department of Precision Medicine, University of Campania “L. Vanvitelli”, 80138 Naples, Italy; 7 Radiology Department, “S. Timoteo” Hospital, 86039 Termoli, Italy; 8 Unit of Diagnostic and Interventional Neuroradiology, 84131 Salerno, Italy

**Keywords:** low-back pain, magnetic resonance imaging, imaging phenotype, radicular pain, vertebrogenic pain, modic changes, image-guided spine interventions, epidural steroid injection, radiofrequency ablation, basivertebral nerve ablation

## Abstract

Low-back pain (LBP) is a leading cause of disability worldwide. When symptoms persist beyond 4–6 weeks, when red flags are suspected, or when precise patient selection for procedures is needed, imaging—primarily MRI (Magnetic Resonance Imaging)—becomes pivotal. The purpose is to provide a pragmatic, radiology-first roadmap that aligns an imaging phenotype with anatomical targets and appropriate image-guided interventions, integrating MRI-based phenotyping with image-guided interventions for subacute and chronic LBP. In this narrative review, we define operational MRI criteria to distinguish radicular from non-radicular phenotypes and to contextualize endplate/Modic and facet/sacroiliac degenerative changes. We then summarize selection and technique for major procedures: epidural and periradicular injections (including selective nerve root blocks), facet interventions with medial branch radiofrequency ablation (RFA), sacroiliac joint injections and lateral branch RFA, basivertebral nerve ablation (BVNA) for vertebrogenic pain, percutaneous disc decompression, minimally invasive lumbar decompression (MILD), and vertebral augmentation for painful fractures. For each target, we outline preferred and alternative guidance modalities (fluoroscopy, CT, or ultrasound), key safety checks, and realistic effect sizes and durability, emphasizing when to avoid low-value or poorly indicated procedures. This review proposes a phenotype-driven reporting template and a care-pathway table linking MRI patterns to diagnostic blocks and definitive image-guided treatments, with the aim of reducing cascade testing and therapeutic ambiguity. A standardized phenotype → target → tool approach can make MRI reports more actionable and help clinicians choose the right image-guided intervention for the right patient, improving outcomes while prioritizing safety and value.

## 1. Introduction

LBP remains the leading global cause of disability, affecting up to two-thirds of adults during their lifetime and with major socioeconomic costs. While many acute episodes resolve spontaneously, subacute and chronic presentations often persist, particularly in degenerative structural changes and/or nerve root involvement [1]. From a pathophysiological standpoint, LBP may start peripherally through inflammatory mediators, neuropeptides, and nerve growth factor, which sensitize nociceptors in the annulus and vertebral endplates and amplify signalling via the sinuvertebral nerve and dorsal root ganglion (DRG), with chronicity contributing to central sensitization [2]. This supports a pragmatic clinical distinction of mechanical versus non-mechanical drivers, and of discogenic/vertebrogenic, neuropathic/radicular, facetogenic, and osseous phenotypes. A key early stratification step is lumbosacral radicular versus non-radicular pain: the radicular phenotype is a dermatomal radiating pain due to nerve-root irritation or compression, whereas radiculopathy implies additional objective neurologic deficits with an annual prevalence of approximately 10–25%, and peak incidence between 45 and 64 years [3]. In this context, radiologists have a dual role—diagnostic role through MRI/CT (Computer Tomography) imaging, with phenotype-oriented imaging assessment, radicular vs. non-radicular stratification, and red-flag exclusion, and interventional role through targeted, minimally invasive image-guided procedures for diagnostic confirmation and symptom relief, by selecting the most appropriate modality (Ultrasound/fluoroscopy/CT/MRI) to optimize visualization and safety for a given anatomical target [4].

Because degenerative MRI findings are prevalent in asymptomatic individuals, imaging must be contextualized: only a minority of primary-care patients require advanced imaging at presentation, and early non-indicated MRI may increase downstream procedures and analgesic medication use without improving outcomes. Therefore, MRI is generally reserved for red flags or persistent symptoms after 4–6 weeks, integrated with clinical evaluation to direct conservative care versus image-guided interventions [5].

For standardized clinical stratification, the BACPAC (Back Pain Consortium) consortium [6] defines chronic LBP as pain present on >50% of days over the prior 6 months and persisting >3 months, providing a shared framework for trials and care pathways.

## 2. MRI Phenotypes of Low-Back Pain

### 2.1. Radicular Pain

Radicular pain arises from inflammation and/or compression of lumbosacral nerve roots, most commonly due to disc herniation or spinal stenosis. MRI correlates include disc–nerve conflict with lateral-recess compromise (e.g., posterolateral protrusion/extrusion impinging the traversing root), foraminal or far-lateral disease affecting the exiting root, and multilevel central canal or lateral-recess stenosis; supportive signs include nerve root calibre change, T2 hyperintensity, and periradicular edema or enhancement [7]. Chemical radiculitis can occur without frank compression, but imaging–symptom discordance is not uncommon: in the clinical practice concordance between the imaging side/level and the patient’s dermatomal pain pattern, sensory deficit, motor weakness, or reflex asymmetry strengthens attribution. Clinically, this phenotype supports targeted image-guided strategies such as epidural steroid injection (ESI) or selective nerve root block (SNRB) for diagnostic and therapeutic decision-making [3].

### 2.2. Facetogenic Pain

Facetogenic pain arises from facet arthropathy, with joint-space narrowing, osteophytes, subchondral change and peri-articular edema/synovitis; however; these features are nonspecific, and MRI may show degenerative joint changes, so diagnostic blocks remain crucial, especially controlled medial branch blocks remain the adjudicator before ablation [8].

### 2.3. Discogenic Pain

Discogenic pain reflects a degenerative cascade in which the intervertebral disc is often the earliest failing structure. Progressive dehydration and loss of disc height, annular fissuring, and aberrant nociceptor/vascular ingrowth can sensitize the annulus–endplate complex and generate pain even in the absence of frank herniation [9].

These changes are further promoted by inflammatory mediators (TNF-α, substance P). Biomechanically, because the annulus is stiffer peripherally, failure tends to occur posterolaterally, mirroring the posterolateral fissures and high-intensity zone (HIZ) patterns encountered on MRI; with time, secondary facet and vertebral remodelling may contribute to canal/foraminal narrowing and predispose to degenerative spondylolisthesis [10].

Clinically, discogenic pain often overlaps with other mechanical and radicular syndromes, so MRI findings such as annular fissures and Modic type 1–3 endplate changes should be interpreted in strict clinic–imaging correlation [9].

### 2.4. Vertebrogenic Pain

Vertebrogenic pain arises primarily from the vertebral endplates, which are richly innervated by the basivertebral nerve; histologic and immunologic evidence supports the endplates as primary pain generators, distinct from discogenic sources centred on the nucleus–annulus complex [11]. Although biological overlap exists due to disc degeneration and annular fissuring that may injure endplates and facilitate nociceptor/vascular ingrowth, the vertebrogenic phenotype is most consistently captured on MRI by Modic type 1–2 changes at the endplate–disc interface, with type 1 reflecting active inflammatory change, sometimes coexisting with annular fissure/high-intensity zone [12].

These findings have practical implications for selection: patients with chronic axial LBP and concordant Modic 1–2 changes at symptomatic levels may be considered for BVNA after adequate conservative therapy and exclusion of competing generators (facet, sacroiliac, radicular). Beyond single markers, the Mo-Fi-Disc score (Modic changes, paraspinal fatty infiltration, and disc degeneration) [13] has been proposed to stratify risk of more severe LBP, supporting multidomain phenotyping and prognostication.

### 2.5. Sacroiliac Joint (SIJ) Pain

On MRI, degenerative SIJ findings are typically supportive rather than diagnostic, including joint space narrowing, capsular/synovial thickening, subchondral sclerosis, and periarticular bone marrow edema on the sacral or iliac sides. Because these signs are nonspecific, they require strict clinic-imaging correlation. Coronal STIR (Short Time Inversion Recovery) sequence increases sensitivity for inflammatory or edema-related changes, can raise pretest probability in buttock-predominant pain, and helps exclude common mimics (e.g., hip osteoarthritis, gluteal tendinobursitis). When clinical suspicion remains high, a small-volume, contrast-confirmed intra-articular anesthetic block is generally regarded as the reference diagnostic test to confirm the SIJ as the dominant pain generator and to inform subsequent treatment selection [7,14].

### 2.6. LF-Driven Lumbar Stenosis

On routine lumbar MRI, hypertrophy of the ligamentum flavum (LF) with posterior epidural fat effacement and dorsal canal crowding supports a dorsal-element stenosis phenotype, often coexisting with facet arthropathy and disc bulge and clinically associated with neurogenic claudication [15]. Axial T2 images best depict central canal narrowing and lateral-recess compromise; redundant cauda equina or root clumping, when present, further supports functional relevance [16]. A pragmatic stenosis report should grade central and foraminal narrowing and may include dural-sac cross-sectional area thresholds as supportive anchors. Because imaging severity and symptoms can diverge, these findings should be interpreted in strict clinical correlation [17].

### 2.7. Osseous Pain

Osseous pain due to fracture, infection, or malignancy should be treated as a red-flag phenotype: marrow edema and enhancement patterns must be interpreted in the clinical context to avoid mislabeling specific pathology as degenerative LBP. In these cases, imaging primarily supports urgent triage and etiologic work-up rather than phenotype-driven degenerative interventions [2].

## 3. From MRI Phenotype to Targeted Intervention

In subacute and chronic LBP, imaging should follow the clinical approach, which prioritizes triage, phenotype identification, and actionable targeting while minimizing low-yield testing. Imaging should be interpreted as an enabler of clinic–radiologic concordance rather than a standalone diagnostic arbiter [4].

Urgent MRI is primarily indicated for severe or progressive neurologic deficits, or when cauda equina syndrome, infection, or malignancy are suspected.

Radiographs remain appropriate when compression fracture or inflammatory spondyloarthropathy is a concern, with MRI reserved for equivocal, persistent, or clinically complex cases. CT and CT myelography retain a complementary role when MRI is contraindicated or limited by hardware-related artefacts, and CT is particularly clear for osteophytes, vacuum phenomenon, and calcifications that may influence stenosis attribution and procedural planning. In nonspecific LBP, early non-indicated imaging may increase downstream healthcare utilization without improving outcomes, supporting appropriateness-based deployment of advanced imaging [6].

MRI remains the cornerstone for phenotyping because it can integrate disc morphology, posterior element arthropathy, epidural lipomatosis, and mixed contributors to stenosis and pain-generator assessment that helps route patients toward conservative care, image-guided interventions, or surgical evaluation when appropriate [3].

A pragmatic non-contrast MRI protocol typically includes sagittal T1- and T2-weighted imaging, sagittal STIR to assess marrow edema, and axial T2-weighted imaging targeted to symptomatic levels; while contrast-enhanced imaging should be reserved for selected scenarios (e.g., suspected infection, tumour, or postsurgical complications) [10].

For persistent radicular symptoms, a coronal STIR can be particularly helpful to detect extraspinal mimics and relevant co-pathologies (e.g., hip osteoarthritis, gluteal tendinobursitis, sacroiliac arthropathy) that may otherwise confound phenotype attribution [7].

When MRI shows multiple plausible pain generators or nonspecific degenerative findings, targeted diagnostic blocks can help confirm the dominant symptomatic source prior to definitive therapy, reducing overtreatment [18].

Accordingly, reporting should comprehend the most likely phenotypes, the actionable targets and laterality/levels, red-flag mimics, and recommend the next most appropriate steps, thereby reducing misinterpretation and cascade testing [2].

Phenotyping should document Modic/endplate changes, disc degeneration patterns, facet and sacroiliac features, and stenosis descriptors, while explicitly acknowledging that single imaging markers are not diagnostic in isolation and must be contextualized [13]. When MRI is inconclusive or contraindicated, emerging techniques such as ultrashort echo-time MRI and SPECT/CT (Single Photon Emission Computed Tomography) can improve endplate depiction and, in selected circumstances, may support decision-making in carefully chosen patients [19].

Standardized nomenclature and reproducible grading systems should be used to support clarity and interdisciplinary communication, while maintaining the primacy of clinical correlation [10]. Harmonized datasets and protocols can enhance reproducibility across sites and facilitate consistent phenotyping and outcome assessment [5].

Interventional outcomes are generally most favourable when selection is mechanism- and phenotype-driven rather than imaging-driven alone, and when procedures are framed as both diagnostic and therapeutic tools within an integrated pathway [20].

Finally, guidance modality should be chosen pragmatically according to anatomy, safety, and feasibility: fluoroscopy supports real-time needle control, CT offers high spatial resolution for osseous and deep targets, MRI guidance may be useful in selected scenarios [3]. Ultrasound can improve safety when soft-tissue visualization and Doppler mapping are advantageous, probe selection should match target depth and procedural intent [21].

## 4. Interventional Options for Radicular Pain

Radicular pain may warrant image-guided intervention when dermatomal symptoms persist beyond 6 weeks, despite appropriate conservative management.

Accordingly to the clinic-radiologic concordance, MRI evidence of disc–root conflict and/or supportive inflammatory signs should align with dermatomal distribution and neurologic findings before proceeding [3].

In this setting, epidural steroid injections (ESIs) aim to reduce inflammatory radiculitis and provide short-term relief. When the symptomatic level is uncertain in multilevel disease, selective nerve root block (SNRB) may help localize the pain generator. In refractory cases or when steroid exposure is undesirable, pulsed radiofrequency (PRF) of the dorsal root ganglion can be considered as a non-destructive neuromodulatory option, acknowledging heterogeneous evidence [17].

### 4.1. Epidural Steroid Injections

ESIs deliver local anesthetic and corticosteroid into the epidural space via interlaminar (IL), transforaminal(TF) [22], or caudal routes selected according to anatomy and laterality [23]. Overall, ESIs provide modest, short-term benefit for radiculopathy due to disc herniation, while evidence is less consistent in lumbar spinal stenosis [17].

IL-ESI provides broader posterior epidural spread, useful for central/bilateral symptoms, while TF-ESI provides more selective ventral delivery at the symptomatic foramen, and caudal ESI is typically reserved for postsurgical or technically challenging anatomy but is less selective and requires larger volumes [22].

Safety considerations should be stated: TF injections carry higher risk if inadvertent intravascular injection occurs [23]; therefore, image guidance (fluoroscopy or CT; ultrasound in selected cases) and contrast confirmation are commonly used [4], and non-particulate steroids are generally preferred for TF-ESI due to rare catastrophic ischemic complications linked to particulate agents [20].

Consensus statements support TF-ESI for disc-herniation radiculopathy more strongly than for stenosis, and caudal approaches are often less effective than TF in comparative pathways [24]; however, some CT-guided cohort data report similar improvements in pain and disability with TF and IL approaches and low complication rates, suggesting IL-ESI can be a reasonable alternative in selected patients [25].

Notably, short-term TF-ESI outcomes may be comparable with or without visible disc–root contact on MRI, supporting inflammatory radiculitis as a relevant target and discouraging use of “MRI contact” as a strict gatekeeper [26].

Anthropometric surrogates such as MRI-derived subcutaneous fat index should not guide selection because they do not predict response [27]. Intradiscal steroid injection targets axial discogenic pain rather than radicular inflammation and is not recommended routinely for chronic non-radicular LBP in major guidance [28]. Procedural efficacy and safety depend on accurate anatomic understanding across cross-sectional imaging and fluoroscopy [29].

### 4.2. Selective Nerve Root Blocks

SNRB is primarily a diagnostic localizer when multiple levels are plausible [17]: concordant, time-limited relief after a correctly placed small-volume anesthetic supports level selection for surgery or targeted interventions, while any therapeutic effect is secondary [18]. A key limitation is epidural spread to adjacent levels (false positives), so results must be interpreted in conjunction with imaging and examination; routine deep sedation should be avoided to preserve symptom feedback [30].

### 4.3. Pulsed Radiofrequency of the Dorsal Root Ganglion

DRG-PRF may be considered for selected refractory radicular pain, particularly when ESIs provide only transient benefit or steroid exposure is undesirable [31]. PRF applies short RF bursts maintaining non-ablative temperatures (typically <42 °C) to modulate DRG excitability; precise placement under fluoroscopy/CT with sensory and motor testing is recommended [32].

Complications are uncommon but include transient neuritic pain, bleeding, infection, and rare equipment-related burns; many practices limit frequency) [32]. Evidence remains mixed: some analyses report modest short-term advantage over epidural strategies without consistent long-term functional gains, supporting shared decision-making and realistic expectations [31]. Conversely, in selected cohorts, PRF added to TF-ESI has been associated with greater improvements up to 52 weeks versus TF-ESI alone, whereas adding immediate TF-ESI after PRF has not shown additional benefit at 1–3 months [33].

Overall, DRG-PRF is best positioned as a steroid-sparing, adjunct or second-line option with short- to mid-term benefit in selected patients, with durable outcomes still evolving [34]. Periforaminal oxygen–ozone injection is an emerging approach often used when discogenic and radicular components coexist, but current data are sparse and largely short-term, so it should be framed as investigational/context-dependent [31].

A concise target–tool match is provided in Table 1.

## 5. Interventional Options for Non-Radicular Pain

### 5.1. Facetogenic Pain

Facet-mediated pain typically presents as axial LBP worsened by extension and rotation, while MRI/CT findings of facet arthropathy and synovial cysts are supportive rather than diagnostic. Diagnosis is primarily clinical–functional and should be confirmed with controlled medial branch blocks (MBBs) performed using local anesthetic and minimal/no sedation to reduce false positives [24]. A clinically meaningful response supports facetogenic pain and further treatment of denervation, but response thresholds vary across pathways and study designs. Medial branch radiofrequency ablation is best positioned after positive MBBs, and MBBs remain the most reliable prognostic test for RFA success [32]. Notably, a subset of patients may experience prolonged relief after MBB alone, supporting reassessment before proceeding to RFA in selected cases [8].

Intra-articular facet steroid injections are mainly diagnostic/short-term and should not be used as stand-alone definitive therapy [32].

From a procedural standpoint, accurate targeting is emphasized across technical literature [16]. Standard fluoroscopic acquisition (true lateral and true AP) improves reproducibility and documentation and may reduce variability across operators and settings [31,33]. Ultrasound-guided facet injection has been reported as safe and cost-effective with outcomes comparable to fluoroscopy/CT in suitable candidates, but accuracy may decline in obesity or spondylolisthesis [34,35].

MRI-guided lumbar facet denervation has shown technical feasibility and short- to mid-term pain reduction in early series, with emerging interest in targeting refinements and alternative cannula designs for challenging anatomy [22,36].

Higher responder rates are reported when technique parameters are optimized (e.g., parallel orientation, larger cannulae/active tips) [37]. Perpendicular multi-tined approaches have shown clinically meaningful improvements in selected cohorts, with functional predictors of response such as regular exercise and active employment [38].

In the recent literature, it is reported that, in chronic facet-mediated pain refractory to conservative care, mid-term outcomes are generally improved in RFA over steroid injection in appropriately selected patients [37]. Serious adverse events are uncommon when anticoagulation is managed and sterile, image-guided technique is applied [33].

Delphi consensus also supports image guidance over blind approaches and reports comparable outcomes between facet joint injections and MBBs, often without added corticosteroid [32].

**Figure 1:**
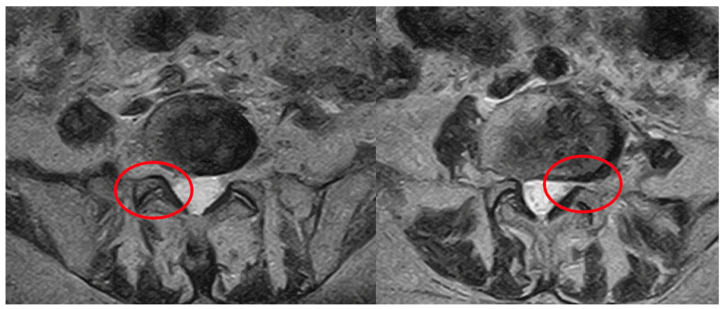
Disc-osteophyte complex with predominant right paramedian extension at the L5–S1 intervertebral level. Based on clinical and neuroimaging findings, an intradiscal ozone injection at L5–S1 and a right-sided facet injection (red circle) with analgesic block at L3–L4 and L4–L5 are proposed, with subsequent reassessment for targeted radiofrequency treatment of the L3–L4 and L4–L5 facet joints.

Figure 1 provides an illustrative case of mixed degenerative findings where a staged diagnostic-to-therapeutic approach is proposed.

### 5.2. Sacroiliac Joint Pain

The SIJ may account for a meaningful proportion of LBP, yet no single clinical or imaging feature is diagnostic [24]. Clinical probability increases with buttock-predominant pain below L5, Fortin area tenderness, and clusters of positive provocation tests, but confirmation is best achieved with an image-guided small-volume intra-articular block [24]. Under fluoroscopy or CT, contrast confirmation and low total injectate volume are used to limit extra-articular spread. Marked immediate pain reduction after local anesthetic injection supports SIJ dominance, while thresholds vary across studies and clinical protocols.

Therapeutically, corticosteroid injections may provide short-term relief, whereas longer durability in block-confirmed responders is more consistently reported with radiofrequency denervation of sacral lateral branches (S1–S3) and the L5 dorsal ramus [17].

Cooled systems may generate larger, more contiguous lesions and are commonly used to address the variable lateral-branch network. Comparative and randomized data report meaningful pain and functional improvement, and selection may be strengthened by a positive prognostic lateral-branch block prior to ablation [16]. Ultrasound-guided SIJ injection is feasible in favourable acoustic windows, but fluoroscopic/CT confirmation remains advisable when landmarks are uncertain [21]. More invasive alternatives (e.g., endoscopic rhizotomy) should be framed as salvage options supported mainly by nonrandomized evidence despite promising matched comparisons [38].

### 5.3. Discogenic Pain

Disc-related pain encompasses contained disc herniation with radicular features, and axial discogenic pain from annular/endplate disease, with different targets and expected effect sizes [9].

In the first case, selected patients with contained disc herniation and concordant radicular symptoms persisting after 4–6 weeks of conservative care, percutaneous disc decompression may be considered to reduce intradiscal pressure and improve symptoms [39].

Available techniques include thermal (laser, radiofrequency, coblation), chemical nucleolysis (oxygen–ozone, gelified ethanol), and mechanical debulking systems [40].

For predominantly inflammatory radicular pain, ultrasound-guided periforaminal oxygen–ozone has been proposed as a strategy with early benefits in selected settings [31]. While improvement rates are often favourable, high-quality evidence remains limited. Complications are uncommon but include spondylodiscitis, reinforcing the need for meticulous sterile technique and imaging guidance [16,30].

Percutaneous Laser Disc Decompression (PLDD) is a promising option with generally minor transient adverse events, though overall evidence quality remains limited; single-centre retrospective data report superior 6-month pain relief compared with conservative care in selected patients [40,41].

Percutaneous endoscopic lumbar discectomy (PELD) is a minimally invasive decompressive approach to relieve nerve-root compression in contained herniation; leg pain often improves, but residual back pain may persist [42]. Early data suggest that adjunctive sinuvertebral nerve ablation may improve LBP outcomes compared with PELD alone, but this remains investigational [43].

In axial discogenic pain, MRI findings such as annular fissures and Modic changes can support phenotyping but are not diagnostic in isolation [10]. Minimally invasive options such as cooled RF (radiofrequency) targeting the annulus show modest, selection-dependent benefit and may be considered in carefully phenotyped cases [9]. Intradiscal steroid injection may provide short-term improvement and help confirm an anterior-column generator but is not supported for routine use and should not replace phenotype-matched strategies [28]. Other techniques (IDET, PIRFT, intradiscal PRF, nucleoplasty) and adjuncts (ozone, methylene blue, biologics) remain investigational with mixed or limited data [10].

Platelet-Rich Plasma (PRP) or mesenchymal stem cells may be considered only in highly selected refractory discogenic pain after multimodal conservative therapy, given low-quality and heterogeneous evidence [24].

**Figure 2:**
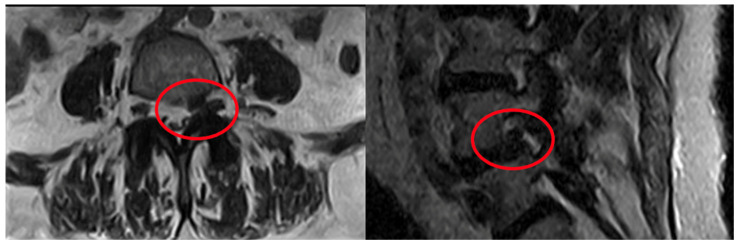
Patient scheduled for oxygen–ozone chemonucleolysis and Periradicular/perivertebral injection: intradiscal O_2_–O_3_ mixture followed by targeted periradicular/perivertebral infiltration (red circles).

Figure 2 provides an example procedural plan for oxygen–ozone chemonucleolysis with adjunct infiltration.

### 5.4. Vertebrogenic Pain (Endplate Nociception)

Vertebrogenic pain is attributed to nociception within the vertebral endplate–marrow unit and is most often associated on MRI with Modic type 1–2 changes used pragmatically to phenotype candidates [23]. Because Modic changes can also occur in asymptomatic individuals, clinic–imaging correlation and exclusion of competing generators remain essential (facet, SIJ, radicular sources) [2]. For persistent axial pain with concordant Modic 1–2 changes after failed structured conservative care, BVNA is an image-guided option with clinically meaningful improvements reported in well-selected patients [24].

Candidate selection should exclude stenosis-dominant presentations, instability, active infection or malignancy, and acute/subacute painful compression fracture, and contraindications (e.g., coagulopathy, pregnancy, skeletal immaturity, severe stenosis, prior surgery at the index level, predominant radicular pain) should be stated explicitly [13].

Under fluoroscopic or CT guidance, transpedicular intraosseous access is used to position the RF probe within the posterior vertebral body adjacent to the basivertebral nerve [44]. Although BVNA is generally safe, rare significant complications (e.g., extradural hematoma likely related to basivertebral venous injury) highlight the importance of antithrombotic management and post-procedure vigilance [45].

### 5.5. Lumbar Stenosis with Hypertrophic LF

Minimally invasive lumbar decompression (MILD) is intended for neurogenic claudication attributable predominantly to hypertrophied ligamentum flavum after failure of conservative care, in the absence of dynamic instability or a competing dominant driver [46]. Performed under fluoroscopy via a small translaminar portal, MILD debulks thickened LF enlarge to the dorsal canal and reduce root crowding. Improvements in pain and function is reported, with durability extending to several years in selected cohorts [47]. Safety profiles appear favourable when selection is appropriate, and contraindicating phenotypes (e.g., high-grade spondylolisthesis/instability or non-LF-predominant stenosis) should be clearly stated; rehabilitation and functional outcome tracking should remain part of the pathway [15,24].

## 6. Osseous Red-Flag Pain

Vertebral augmentation (VA), vertebroplasty (percutaneous PMMA injection) and kyphoplasty (balloon cavity creation followed by PMMA), is best framed as an intervention for painful osteoporotic vertebral compression fractures (VCFs) with imaging evidence of an “active” lesion (MRI marrow edema or uptake on bone scan/SPECT/CT) after failure of conservative therapy [48].

**Figure 3:**
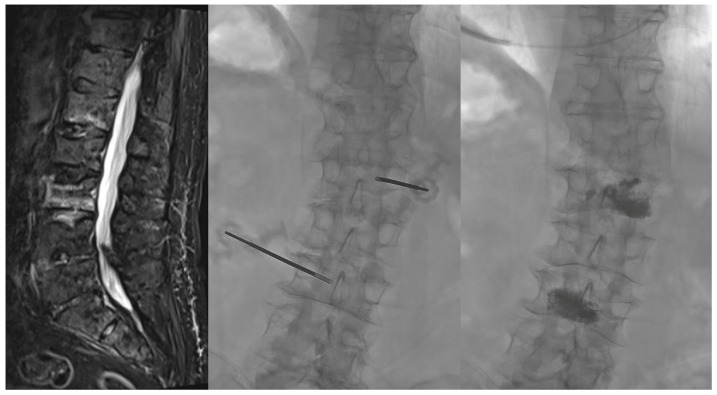
Unipedicular left transpedicular access to the L3 vertebral body and right transpedicular access to L1 for placement of 13-gauge needles. Vertebroplasty was performed with injection of 4 mL PMMA per vertebra.

Vertebroplasty is typically performed via transpedicular access under fluoroscopy guidance or CT for complex anatomy, with reported meaningful pain reduction in appropriately selected VCFs, and with no consistent overall superiority of vertebroplasty versus kyphoplasty [49]. After kyphoplasty, residual LBP may relate more with bone quality, postoperative kyphosis, and paraspinal muscle composition than with cement metrics, supporting a broader biomechanical/functional perspective beyond the procedure itself [50]. In neoplastic vertebral disease, ablation-plus-cement strategies extend the same principles and can yield substantial pain/opioid reduction with expected rates of largely asymptomatic leakage [51]. Figure 3 provides an illustrative vertebroplasty example.

Figure 3 shows a sample of unipedicular vertebroplasty.

## 7. Conclusions and Future Directions

This review rationalizes imaging in subacute and chronic low-back pain by linking MRI phenotype to anatomical target and to interventional tool, across radicular and non-radicular presentations. When symptoms persist beyond guideline-based conservative care, MRI-based phenotyping can support targeted, image-guided interventions only when clinic–imaging concordance is present.

Across all procedures, safety and governance should be treated as core outcomes. Standardized operator training, contraindication screening (including antithrombotic management), sterile technique, appropriate image guidance (with contrast confirmation when indicated), and structured follow-up are essential to reduce preventable complications and to make reported effectiveness credible [21]. Injections should be considered as enablers of functional recovery rather than stand-alone endpoints.

For disc access, vertebral augmentation, and neuromodulation, the IPSIS consensus provides practical safeguards [52]. Clinically, epidural/periradicular injections typically yield short-term relief in radiculopathy, while diagnostic blocks (SNRB, MBB, SIJ) help arbitrate the dominant generator before definitive therapies; medial branch RFA and BVNA can offer more durable benefit in selected facetogenic and vertebrogenic phenotypes. Vertebral augmentation should remain anchored to the osseous phenotype of an active painful osteoporotic compression fracture rather than degenerative chronic LBP. Regenerative biologics (e.g., PRP) remain promising but are not yet routine given heterogeneity in preparation, dosing, and trial quality [51]. Future priorities should include pragmatic selection algorithms, with solid comparative effectiveness, and long-term durability for newer techniques.

As a brief AI note, radiomics and machine learning tools are increasingly proposed for prognosis and response prediction in chronic or oncologic settings [53,54,55,56].

Early models have shown potential for predicting residual pain after vertebral augmentation [57]. In endoscopic discectomy, models predict reoperation using recurrent predictors such as BMI, Modic changes, herniation type, and motion/facet features [58,59]. Similar ML frameworks have been reported for PEID/PELD, but prospective external validation and harmonized endpoints remain prerequisites for clinical adoption [60,61].

## Figures and Tables

**Table 1:** Radicular Phenotype: Matching Target and Tool.

Procedure	Target	Key Point
IL ESI	Posterior epidural space	Broader spread; useful for central/bilateral symptoms.
TF ESI	Ventral epidural/foraminal region	Most selective; use image guidance + contrast; non-particulate steroid preferred for safety.
Caudal ESI	Caudal epidural space	Option in postsurgical/limited access; less selective, larger volumes.
SNRB	Exiting/traversing root	Diagnostic localizer in multilevel disease (short-lived concordant relief).
DRG-PRF	DRG	Steroid-sparing neuromodulation; evidence heterogeneous; selection critical.

## Data Availability

No new data were created or analyzed in this study. Data sharing is not applicable to this article.

## Abbreviations

The following abbreviations are used in this manuscript:

AI	Artificial Intelligence
BACPAC	Back Pain Consortium (BACPAC Research Program)
BVNA	Basivertebral Nerve Ablation
DRG	Dorsal Root Ganglion
ESI	Epidural Steroid Injection
HIZ	High-Intensity Zone
IDET	Intradiscal Electrothermal Therapy
IL	Interlaminar
IPSIS	International Pain and Spine Intervention Society
LBP	Low-Back Pain
LF	Ligamentum Flavum
MBB	Medial Branch Block
MILD	Minimally Invasive Lumbar Decompression
ML	Machine Learning
PEID	Percutaneous Endoscopic Interlaminar Discectomy
PELD	Percutaneous Endoscopic Lumbar Discectomy
PIRFT	Percutaneous Intradiscal Radiofrequency Thermocoagulation
PLDD	Percutaneous Laser Disc Decompression
PMMA	Polymethylmethacrylate
PRF	Pulsed Radiofrequency
PRP	Platelet-Rich Plasma
RF	Radiofrequency
RFA	Radiofrequency Ablation
SNRB	Selective Nerve Root Block
STIR	Short Time Inversion Recovery
TFESI	Transforaminal Epidural Steroid Injection
VA	Vertebral Augmentation
VCF	Vertebral Compression Fracture
